# A Study on Tissue-Specific Metabolite Variations in *Polygonum cuspidatum* by High-Resolution Mass Spectrometry-Based Metabolic Profiling

**DOI:** 10.3390/molecules24061058

**Published:** 2019-03-18

**Authors:** Zhijun Wu, Xiaowei Wang, Mo Chen, Hongyan Hu, Jie Cao, Tuanyao Chai, Hong Wang

**Affiliations:** 1 College of Life Sciences, University of Chinese Academy of Sciences, Yuquan Road, Beijing 100049, China; wuzhijun14b@mails.ucas.ac.cn (Z.W.); wangxiaowei15@mails.ucas.ac.cn (X.W.); chenmo215@mails.ucas.ac.cn (M.C.); huhongyan16@mails.ucas.ac.cn (H.H.); caojie17@mails.ucas.ac.cn (J.C.); 2 School of Life sciences and Biotechnology, Heilongjiang Bayi Agricultural University, Daqing 163319, China; 3 Institute of Genetics and Developmental Biology, Chinese Academy of Sciences, Beichen west Road, Beijing 100101, China

**Keywords:** *Polygonum cuspidatum*, LC-MS, metabolomics, multivariate analysis

## Abstract

*Polygonum cuspidatum* Sieb. et Zucc. is a traditional Chinese herbal medicine widely used to treat tussis, hepatitis and arthralgia. This study identified and quantitatively described the bioactive compounds in different *P. cuspidatum* tissues. Metabolic profiles of root, stem, leaf, flower, rhizome and seed were determined using high-resolution mass spectrometry in combination with multivariate analyses. In total, 53 metabolites, 8 reported for the first time in this species, were putatively identified and classified mainly as stilbenes, anthraquinones and flavonoids. A principal component analysis, cluster analysis and heatmap were used to depict the correlations between specimens and the relative abundance levels of these compounds in different plant tissues. An orthogonal partial least square discriminant analysis found that 13 metabolites showed distinct differences among the six plant tissues, making them potential discriminative tissue-identification markers. This study will provide guidance in comparing, selecting and exploiting the medicinal uses of different *P. cuspidatum* tissues.

## 1. Introduction

*Polygonum cuspidatum* Sieb. et Zucc. is an herbaceous perennial plant of the Polygonaceae family, whose members contain high levels of resveratrol and emodin [1]. The root and rhizome of *P. cuspidatum* have been widely used to treat tussis, hepatitis, jaundice and arthralgia for thousands of years in China and East Asia [2,3]. *P. cuspidatum*’s pharmacological effects result from the presence of large amounts of phenolic compounds, including anthraquinones, stilbenes, flavonoids and isoflavonoids [4]. Phenolic compounds have antioxidant, anti-microbial and anti-carcinogenic characteristics, which are beneficial to human health [5,6]. In addition, the average levels of resveratrol and emodin in *P. cuspidatum* are much greater than in other plants; therefore, they are used as the indicator compounds to characterize the quality of this plant in Chinese Pharmacopoeia. Thus, *P. cuspidatum* is considered the main source of natural resveratrol and emodin [7,8].

According to Chinese Pharmacopoeia, the root and rhizome of *P. cuspidatum* are the main tissues used to make compounds classified as ‘Traditional Chinese Medicines’ in clinical trials [9], while most of the leaves, flowers, stems and other tissues are usually thrown away. The young tender leaves and stems of *P. cuspidatum* are edible and used to treat hepatitis and enteritis by the Yi and Miao ethnic groups in China [10,11]. The phytochemistry and pharmaceutical effects of the root and rhizome of *P. cuspidatum* have been studied [12,13]; however, less is known about the bioactive composition and medicinal values of the other tissues [14]. In addition, the metabolic profiles and chemical differences among different tissues of *P. cuspidatum* have not been elucidated systematically. 

Metabolomics, especially based on liquid chromatography coupled with high-resolution mass spectrometry (LC-MS), has been widely used to investigate the comprehensive profiles of natural products in herbal plants. Ding et al. [15] established a metabolomics approach based on high-resolution LC-MS and chemometric methods for the quality evaluation and discrimination of natural products. They successfully screened, identified and quantified five markers for the precise quality evaluation of crude and carbonized *Pollen Typhae*. Geng et al. [16] developed a powerful strategy based on high-resolution quadrupole time-of-flight (Q-TOF) MS to explore the chemical ingredients and transformation mechanisms in cultivated *Bulbus Fritillariae cirrhosae*. They identified four alkaloid compounds that could be used as potential chemical markers for the classification of cultivated *Bulbus Fritillariae cirrhosae* samples at different growth stages. 

Previously, it was reported that the root and rhizome of *P. cuspidatum* are the main tissues that have pharmacological effects. We suspect some compounds in other tissues may also be beneficial to human health and could be used in clinical trials. In the present study, metabolic profiles of six different tissues; root, stem, leaf, flower, rhizome and seed of *P. cuspidatum*, were determined using high-resolution MS. The bioactive compounds of *P. cuspidatum* were identified, and the relative contents of these compounds among tissues were measured. Statistical analyses confirmed that tissues of *P. cuspidatum* could be distinguished by chemical markers. The results obtained in this study provide valuable insights into the distribution of bioactive compounds in different *P. cuspidatum* tissues and may also provide some practical guidance for choosing the appropriate medicinal tissues for clinical applications. 

## 2. Results and Discussion

### 2.1. Optimization of the Extraction Procedure and MS Conditions

The metabolite extraction procedures are crucial for further metabolomics studies. To extract and detect as many compounds as possible, the extraction parameters, including extraction solvents, extraction time, extraction temperature and extraction repetitions, were optimized. The optimized method is described in Section 2.2. The chromatographic conditions and MS parameters were also optimized. Acetonitrile and water, each supplemented with 0.1% formic acid, were used as mobile phases. The applied gradient was optimized and is described in Section 2.3. Both positive and negative ion modes were performed to determine the suitable ion mode. In negative ion mode, more peaks were detected than in positive ion mode. In addition, a greater sensitivity and clearer structural information were obtained in negative ion mode than in positive ion mode. Therefore, the MS analysis of extracted metabolites was performed in negative ion mode.

### 2.2. Comparison of Metabolic Profiles of Different Tissues

High-resolution Q-TOF MS is an analytical platform that has excellent reproducibility and stability, and is widely used for the identification of metabolites [17]. In the present study, high-resolution LC-Q-TOF MS was used to determine the relative levels and global distribution profiles of metabolites extracted from different tissues of *P. cuspidatum*. The significant differences and chemical markers among tissues were also investigated. Each group of tissue contained six biological replicates, except the rhizome group had five replicates. The total ion chromatograms of each sample group were very similar. XCMS, a metabolomic data processing platform, was used to analyze the raw data, and 4309 aligned features (including unidentified compounds) were detected. Typical LC-MS spectra are shown in Supplemental Figure S1. 

### 2.3. Construction of Predictive Models for the PCA

**Figure 1:**
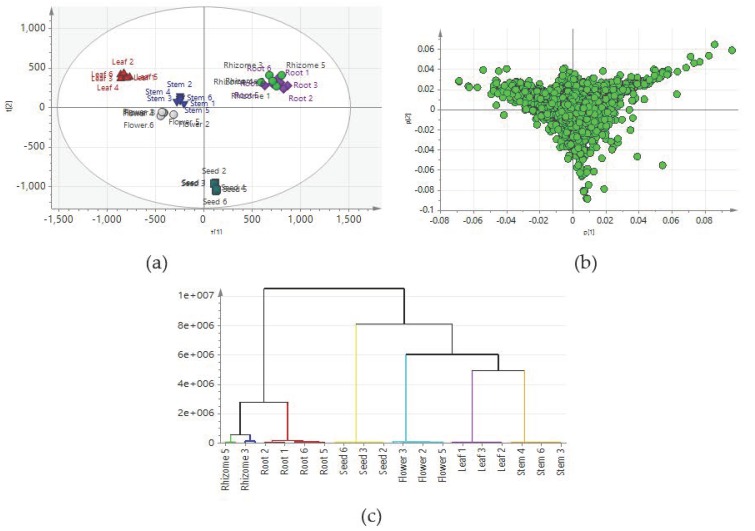
Principal component snalysis (PCA) and hierarchical cluster analysis on root, rhizome, leaf, flower, stem and seed groups. (**a**) Scores plot, (**b**) Loading plot, (**c**) Hierarchical cluster analysis of all samples. PCA and Hierarchical cluster analysis using all of the 4309 ion signals. Scores plot and loading plot of PCA model gives 2D visual information about the variations among different groups. Unsupervised PCA score plots showing the discrimination of samples. R^2^ = 0.741, Q^2^ = 0.621.

Principal component analysis (PCA) is an unsupervised multivariate analysis method for the reduction of complex dataset dimensionality to provide important insights into variations among groups. In the present study, 4309 aligned features were analyzed using a PCA to display the differences among groups. The score plots of the PCA revealed that 31.53% and 17.1% of the variation were explained by PC1 and PC2, respectively. The fitness (R^2^) and prediction power (Q^2^) of this PCA model were 0.741 and 0.621, respectively. The PCA revealed an obvious separation among different tissues, except the root and rhizome groups. Thus, the metabolic constituents of leaves, stems, flowers, seeds, roots and rhizomes differed significantly. The root and rhizome groups clustered together and could not be discriminated through the PCA. Because the root and rhizome have grown underground for many years and accumulated lots of secondary metabolites, their metabolites may differ largely from those of other tissues, and thus, can be discriminated by PC1 (Figure 1). The flower and stem groups were relatively closer than those of other tissues. The chemical profile of the seed group was far from those of other tissues and could be separated by PC2. To determine correlations among the different tissues, the 4309 ion signals detected in this experiment were used in a hierarchical cluster analysis (Figure 1). The PCA and hierarchical cluster analysis showed that samples of different tissues could be discriminated on the basis of LC-MS metabolomics.

### 2.4. Tentative Identification of Bioactive Metabolites in P. cuspidatum

The objective of this study was to identify the major bioactive compounds of *P. cuspidatum* and potential chemical markers that contribute to significant classification differences among tissues. These compounds were identified by comparing their accurate molecular weights, retention times and fragment patterns with those reported in the literature, databases, such as HMDB, METLIN and MassBank, and reference standards, respectively. We first searched the molecular ion’s *m*/*z* values of compounds in *P. cuspidatum* found in KNApSAcK, BATMAN-TCM and other references [1,2,3]. Then, the notably different molecular ions were tentatively identified. The diagnostic ion-filtering strategy for rapid identification and the data mining method were also used based on the methods of Liu et al. and Chang et al. [18,19]. In total, 53 compounds, including stilbenes, anthraquinones, flavonoids, isoflavonoids and other compounds, were annotated from the six tissues. The retention times, molecular ions, classes, MS/MS fragment ions, standard compounds and references for these metabolites are listed in Table 1. Polar compounds were eluted first and the relative nonpolar compounds were eluted last. This trend was in accordance with the elution profile of reverse-phase chromatography [20].

#### 2.4.1. Stilbenes and Their Derivatives

**Figure 2:**
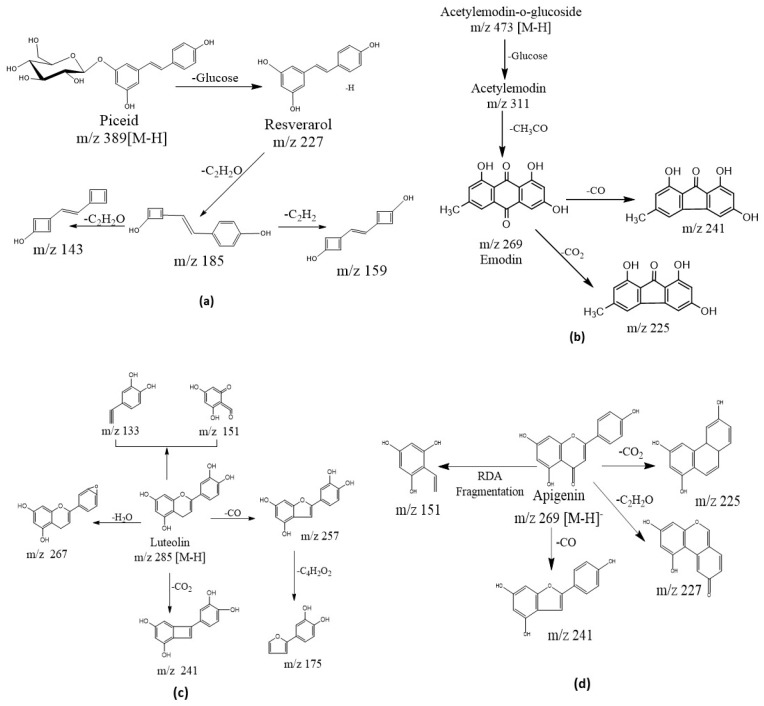
The proposed fragmentation pathways of selected compounds. (**a**) Piceid and resveratrol, (**b**) Acetylemodin-o-glucoside and emodin, (**c**) Luteolin, (**d**) Apigenin. The proposed fragmentation pathways were established based on generated fragment ions.

Stilbenes are the major active components in *P. cuspidatum* and the quality of *P. cuspidatum* is usually evaluated based on their contents. In this study, stilbenes and their derivatives were tentatively identified from different tissues of *P. cuspidatum*. Stilbenes and their aglycones share the same fragment ion at 227 *m*/*z*, the [M − H]^−^ ion of 3,5,4′-trihydroxy-stilbene. For example, the molecular ion M227T7 was eluted at 6.95 min with an *m*/*z* value of 227.0720 ([M − H]^−^), which indicated it was the molecular ion of C_14_H_11_O3. The fragment ions of M227T7 occurred at 185.0624, 159.0824, 143.0506 and 107.0522 *m*/*z* in negative ion mode. Fragments 185.0624 and 143.0506 were formed by successive losses of C_2_H_2_O groups from 227.07 [18]. These fragments and retention times were fitted to the resveratrol standard. Therefore, compound M227T7 was identified as resveratrol. The molecular ions of M389T5 had *m*/*z* values of 389.1236 ([M − H]^−^) and 435.1279 ([M + HCOO]^−^, which corresponded to the molecular formulae of C_20_H_21_O_8_ and C_21_H_23_O_10_, respectively. The ion fragments of M389T5 included a fragment with an *m*/*z* value of 227.0930, which was the same as resveratrol. Thus, a glucoside was cleaved and the precursor ion M389T5 lost a glucose unit. Most resveratrol fragments were detected in the MS/MS analysis of M389T5. The retention time of M389T5 was 5.09 min, which was the same as the piceid reference standard. Therefore, this compound was identified as piceid. The proposed fragmentation pathways for piceid and resveratrol are illustrated in Figure 2.

**Figure 3:**
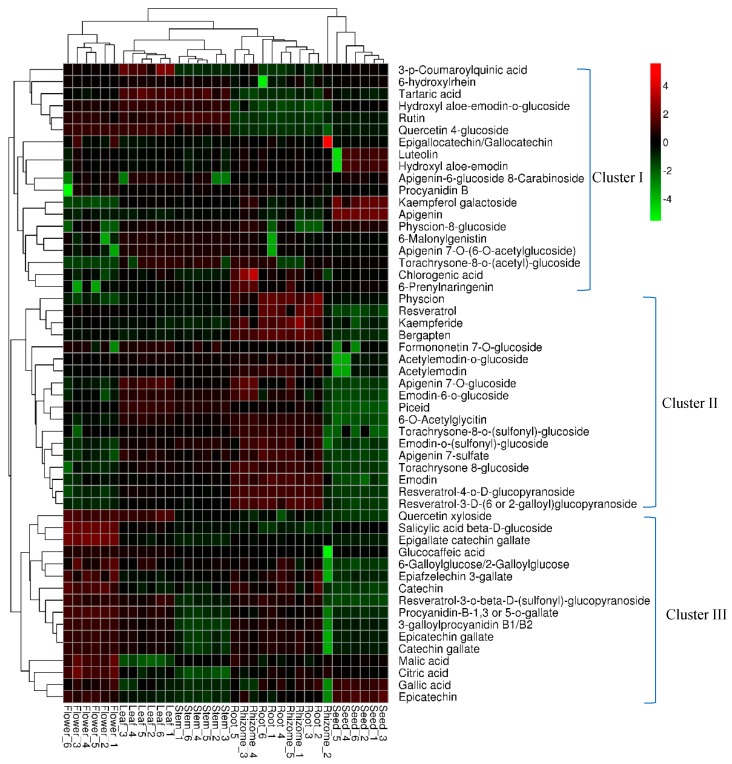
Heatmap analysis of the relative distributions of 53 identified metabolites in different tissues. The heatmap was exhibited using a hierarchical clustering algorithm based on the normalized average signal abundance. The red and green color in the heatmap represent an increase and a decrease of metabolite level, respectively.

The other stilbene compounds displayed the deprotonated [M − H]^−^ and shared the same *m*/*z* 227.0767 fragment. These compounds were tentatively identified by comparing the fragments with the references and databases, including METLIN and HMDB. For example, the ion M541T6 (*m*/*z* 541.1379, retention time 6.20 min) was tentatively identified as resveratrol-3-d-(6-galloyl) glucopyranoside on the basis of the reference [2]. The stilbene contents were significantly greater in root and rhizome than in other tissues (Figure 3), which was consistent with previous studies. In addition, these results support the use of *P. cuspidatum* root and rhizome in ‘Traditional Chinese Medicines’.

#### 2.4.2. Anthraquinones

*P. cuspidatum* is a rich source of anthraquinone and its derivatives, which have many biological activities, such as anti-cancer, anti-inflammatory and anti-methicillin-resistant *Staphylococcus aureus* [26]. In this study, nine anthraquinone compounds, including emodin and its derivatives, were tentatively identified according to the accurate *m*/*z* values (within a mass error of 5 ppm) and characteristic fragments. 1,8-Dihydroxy-9,10-anthraquinone is the common core of anthraquinones and its deprotonation can generate a characteristic fragment ion with an *m*/*z* value of 269.0450. The negative ion MS/MS fragments of M269T15 occurred at 241.0512, 225.0568, 197.0621, 181.0666, 115.0552 and 65.0044 *m*/*z*. The fragments at 241.0521 and 225.0568 *m*/*z* of M269T15 were generated through the loss of CO and CO_2_, respectively. Aloe emodin and emodin are a pair of isomers and have different ion MS/MS fragment distributions. Emodin has a characteristic fragment of 225.05 *m*/*z*, while that of aloe emodin occurs at 239.03 *m*/*z* [18,21]. The retention time of M269T15 was 15.55 min and was consistent with the emodin reference standard. Therefore, M269T15 was identified as emodin. The proposed fragmentation pathways for emodin are shown in Figure 2.

The ion *m*/*z* values of physcion, physcion-8-glucoside and emodin-6-*O*-glucoside were tentatively identified by matching MS/MS spectra with those reported in the literature and databases. M283T15 was identified as physcion, and the precursor molecule’s *m*/*z* value was 283.0611 in the negative ion mode. Its fragments occurred at 268.0362, 240.0412 and 205.0501 *m*/*z*, which are characteristic of physcion. These fragments at 268.0362 and 240.0412 *m*/*z* were generated by the successive losses of a CH_3_ and a CO group [18,21]. The glucosides of physcion were also identified using the above method. For example, M445T10 was identified as physcion-8-glucoside. The MS/MS fragments occurred at 445.1145, 283.0630, 240.0445 and 165.0464 *m*/*z*. Fragment 283.0630 ([M − H]^−^) *m*/*z* represented the ion of physcion, and the fragment at 240.0445 *m*/*z* was characteristic of physcion [27].

#### 2.4.3. Flavonoids, Isoflavonoids and Other Compounds

Flavonoids are a large family of compounds with a variety of bioactive effects, such as antioxidant and anti-inflammatory activities, and play important roles in the coloration of flowers and ripening fruit [28]. In this study, 25 compounds were putatively identified as flavonoids and flavonoid glucosides by comparing the accurate *m*/*z* values and typical fragments with databases, literature and standards.

Compound ion M289T3 was identified as catechin with a precursor molecular [M − H]^−^ ion at 289.0737 *m*/*z*. The MS/MS fragments occurred at 271.0656, 245.0885, 221.0838 and 151.04 *m*/*z*. The retention time of M289T3 (2.64 min) and the fragment mode were the same as those of the reference standard. Epicatechin-gallate and rutin were identified by analyzing the fragments against metabolomics databases and references [23]. Molecular ion M269T9 was tentatively identified as apigenin. The *m*/*z* value of M269T9 was 269.0449, with a retention time of 9.10 min. Fragments of M269T9 occurred at 269.0494, 227.0409, 225.0536 and 151.0063 *m*/*z*. Ions of 227 ([M − H − C_2_H_2_O]^−^) and 225 ([M − H − CO_2_]^−^) were generated by losing a C_2_H_2_O group and a CO_2_ group, respectively. The ion fragment at 151.00 *m*/*z* was formed by the cleavage of the C-ring [29]. There was no vicinal-hydroxyl group in apigenin, but it was not the result of the neutral loss of a H_2_O group. Thus, an ion at 251 *m*/*z* ([M − H − H_2_O]^−^) was not found in the MS/MS spectrum of apigenin [30]. The MS/MS spectrum of M269T9 was in accordance with the fragments of apigenin [25]; therefore, it was tentatively identified as apigenin. Similarly, M349T13 and M431T7 were tentatively identified as apigenin 7-sulfate and apigenin 4-*O*-glucoside, respectively.

Luteolin was also identified by analyzing the fragments of M285T8. The ion fragments *m*/*z* occurred at 285.0381, 267.0342, 257.0439, 243.0279, 241.0489, 229.0493, 217.0488, 175.0381, 151.0031, 133.0329, 121.0273 and 107.0162 *m*/*z*. Luteolin has a vicinal-hydroxyl group, and it can generate ion ([M − H − H_2_O]^−^) at 267 *m*/*z* by losing a H_2_O group. The ions at 257 and 241 *m*/*z* were formed by losing a CO and CO_2_ group, respectively. The two fragments at 151.00 and 133.03 *m*/*z* were generated through retro-Diels-Alder cleavage, which is the characteristic flavonoids pathway [24,31]. With this information on the fragment ions, M285T8 was tentatively identified as luteolin [29,30]. The proposed fragmentation pathways for apigenin and luteolin are illustrated in Figure 2. Other compounds included tannins, such as gallic acid and bergapten. These compounds were also tentatively identified using databases and references. We also identified the compounds luteolin, epigallate catechin gallate, apigenin-6-glucoside, kaempferide, formononetin 7-*O*-glucoside, bergapten, 6′-malonylgenistin and procyanidin-B-1, 3-*O*-gallate for the first time, to our knowledge, in this species.

### 2.5. Global Distribution and Relative Quantitative Analysis of Identified Metabolites

The distribution and relative quantification of the identified metabolites from different tissues of *P. cuspidatum* were illustrated using a heatmap (Figure 3). The heatmap was constructed using a hierarchical clustering algorithm based on area-normalized metabolite quantities. The red and green colors in the heatmap represent higher and lower relative contents than the average value, respectively. In the heatmap, root and rhizome samples formed a group, except for sample “rhizome 2”, which was assigned to the seed group. Other samples from different tissues were clearly separated, suggesting that the metabolites were significantly different among tissues. As described in Figure 3, the relative abundance levels of the 53 compounds from different tissues were divided into three clusters. Cluster I mainly contained flavonoids, including apigenin, luteolin, rutin and apeginin-7-*O*-glucoside. The greater relative contents of these compounds were found in the leaf and stem groups. However, the other compounds, including 6-prenylnaringenin, gingerol, chlorogenic acid, and torachrysone 8-glucoside, were present at greater levels in rhizomes. Apigenin and luteolin are present in a variety of fruit, vegetables and medicinal plants, and play multiple roles in development and plant–environment interactions. Apigenin and its glycosides are present in seeds of *Lupinus*, and apigenin can induce the antioxidant defense system in seedlings [32,33]. In this study, apigenin and luteolin were detected in *P. cuspidatum* and their relative contents in seeds were greater than in other tissues. Apigenin might enhance antioxidant activities in seeds and improve the germination rate of *P. cuspidatum*. Apigenin and luteolin have many beneficial effects on human health, including antitumor, anti-inflammatory, neuroprotective effects, and biological organ protection and immune regulation [34,35]. Thus, these results provide insights into the possible exploitation of *P. cuspidatum* seeds to improve human health. The rutin and quercetin 4-glucoside contents were greater in leaves, stems and flowers than in other tissues. This might explain why the leaves and stems of *P. cuspidatum* are used as medicine and food.

Cluster II mainly contained anthraquinones, stilbenes and other compounds, such as torachrysone 8-glucoside, bergapten and kaempferide. Stilbenes and anthraquinones, including resveratrol, emodin and their derivatives, had much greater abundance levels in roots and rhizomes than in other tissues. This could be the basis of why the root and rhizome of *P. cuspidatum* are categorized as medicinal tissues in Chinese Pharmacopoeia. The concentrations of these compounds in the seeds and flowers were lower than in other tissues. Torachrysone 8-glucoside and torachrysone-8-*O*-(acetyl)-glucoside were either not detectable or present at extremely low levels in flowers and seeds.

Catechin, epicatechin, quercetin xyloside, epigallate catechin gallate, epiafzelechin 3-gallate, 3-galloylprocyanidin B1 or B2 and procyanidin-B-1, 3-*O*-gallate were mainly present in Cluster III. These compounds were present in greater concentrations in flowers than in other tissues. The accumulation of these compounds in flowers could protect the plant from pathogen attacks and abiotic stresses [36,37]. Karakaya reported a similar result, namely that the flowers of *Z. absinthifolia* can be a potential resource of natural antioxidant compounds [38]. The abundance levels of the other compounds in this cluster, except gallic acid and quercetin xyloside, were lower in seeds than in other tissues. These compounds have many benefits to human health. For example, epigallate catechin gallate could be used to treat cyclic GMP-AMP synthase mediated autoimmune diseases [39]. Thus, the flowers of *P. cuspidatum* could be further processed into tea or herbal beverages.

### 2.6. Identification of Chemical Markers

**Figure 4:**
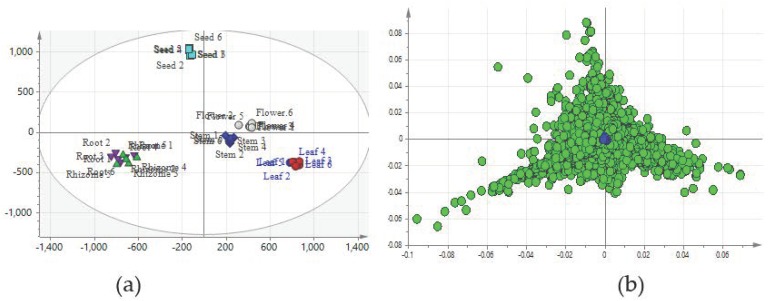
OPLS-DA score plots and loading plots. (**a**) Score plots; (**b**) loading plots. The R^2^X, R^2^Y and Q^2^ values of OPLS-DA were 0.726, 0.987 and 0.973, respectively.

Metabolomics and multivariate statistical analyses, including orthogonal partial least square discriminant analysis (OPLS-DA), have become powerful tools to distinguish the differences among metabolite patterns and reveal marker compounds in different samples [40,41]. Mo et al. used UPLC-MS-based untargeted metabolomics profiling to discriminate samples from different parts of *Panax notoginseng* [42]. Li et al. established an UPLC-QTOF-MS-based metabolomics approach and found eight markers that played significant roles in the bioactivities of branch extracts of *Garcinia oblongifolia* [43]. The roots and rhizomes are the main medicinal parts of *P. cuspidatum* according to Chinese Pharmacopoeia, but the chemical differences among different tissues remains unknown. OPLS-DA was used to investigate the differential compounds among tissue groups. The score and loading plots of OPLA-DA are presented in Figure 4. The score plots and explained variances of the OPLS-DA model were similar to those of the PCA plots. The R^2^X, R^2^Y and Q^2^ values were 0.726, 0.987 and 0.973, respectively. These parameters suggested that the model was a good fit and had a satisfactory predictive capability.

**Figure 5:**
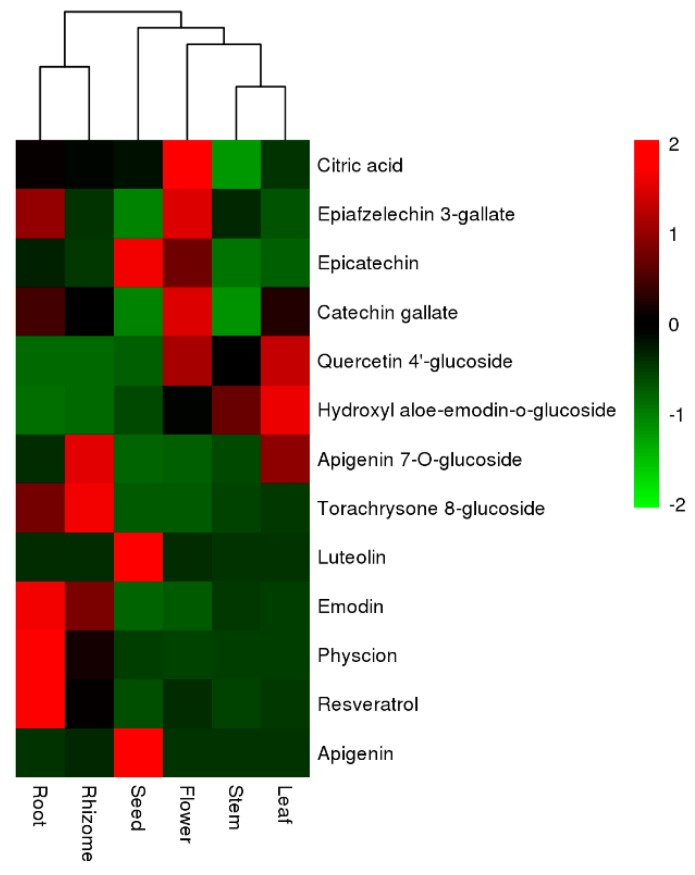
The relative contents of thirteen markers in different tissues of *P. cuspidatum*. The contents of each compound was normalized as a relative content. The heatmap was exhibited using a hierarchical clustering algorithm based on the normalized average abundance.

The potential chemical markers that could distinguish different tissues of *P. cuspidatum* were selected using OPLS-DA and putatively identified. The marker ions were screened according to the OPLS-DA loading plots, the VIP value (>1) and the P value (<0.001), and the following 13 potential chemical marker ions were tentatively identified: emodin, quercetin 4′-glucoside, epiafzelechin 3-gallate, epicatechin, citric acid, luteolin, apigenin, apigenin 7-*O*-glucoside, hydroxyl aloe-emodin-o-glucoside, catechin gallate, torachrysone 8-glucoside, physcion and resveratrol. The relative abundances of these compounds are displayed in Figure 5. The contents of most of the compounds differed significantly among different groups; consequently, they might be used as markers for differentiating tissues of *P. cuspidatum*. The relative contents of emodin, torachrysone 8-glucoside, resveratrol and physcion were as follows: roots/rhizomes > leaves > stems > flowers > seeds. Thus, these compounds could be used to distinguish the root/rhizome from other tissues of *P. cuspidatum*. The greatest contents of luteolin, apigenin and epicatechin were found in seeds and could be used as markers to distinguish seed from other tissues. The citric acid, epiafzelechin 3-gallate and catechin gallate contents were greater in flowers than in other tissues, while those of the hydroxyl aloe-emodin-*O*-glucoside, quercetin 4′-glucoside and catechin gallate were greater in leaves than in other tissues.

The identification of these potential markers provided useful information for the further isolation of functional genes in combination with transcriptome or genome data, and they could be applied to discriminate among the different tissues of *P. cuspidatum*. Our results provide some guidance for the further exploitation of *P. cuspidatum*, in particular the different tissues of this medicinal herb. By combining these results with transcriptome data, it may be possible to identify functional genes correlated with the biosynthesis of pharmaceutical compounds.

## 3. Materials and Methods

### 3.1. Plant Materials and Chemicals

The root, rhizome, stem, leaf, flower and seed of *P. cuspidatum* grown in the Institute of Botany (Beijing, China), the Chinese Academy of Sciences, were harvested for metabolite extraction. Tissues for each biological replicate sample were collected from three individual plants. All samples were frozen immediately and then transferred to −80 °C for storage until analysis.

All chemical standards used in this study were purchased from Sigma-Aldrich (St. Louis, MO. USA). HPLC grade methanol, acetonitrile and formic acid were obtained from Merck (Darmstadt, Germany). Ultrapure water was generated by a Milli-Q system (Millipore, MA, USA).

### 3.2. Metabolite Extractions

Samples with biological replicates were prepared for each tissue group. Tissue samples were ground to a powder using a pre-chilled mortar and pestle, and then freeze-dried. Briefly, 50 mg freeze-dried plant tissue was weighed and transferred into a 2-mL centrifuge tube. Then, 1 mL MeOH–H_2_O (80:20, *v*/*v*) was added, vortexed vigorously three times, and an ultrasonic-assisted extraction was performed for 30 min in an ice bath. Samples were extracted overnight at 4 °C and then centrifuged for 15 min at 12,000× *g*. Afterward, 500 μL clear supernatant per extract was filtered through a 0.22-μm PTFE filter before the LC-MS analysis.

### 3.3. LC-MS Analysis

The metabolomics analysis was performed using an Agilent 1290 HPLC system coupled with a Q-TOF ESI mass spectrometer (Agilent, Santa Clara, CA, USA). HPLC separation was performed using a Phenomenex reversed-phase C18 column (Kinetex, 2.6 μm, 100 mm × 2.1 mm, Phenomenex Inc., Torrance, CA, USA). Water supplemented with 0.1% formic acid (A) and acetonitrile supplemented with 0.1% formic acid (B) were used as mobile phases at a flow rate of 0.4 mL/min. The gradient was optimized as follows: 0–1 min: 5% B; 1–6 min: 5%–23% B; 6–10 min: 23%–29% B; 10–18 min: 29%–70% B; 18–26 min: 70%–98% B; 26–29 min: 98% B; 29–30 min: 98%–5% B; and 30–33 min: 5% B. In all cases, the injected volume was 10 μL, and the column temperature was maintained at 30 °C. An Agilent 6530 high-resolution Q-TOF mass spectrometer (Agilent, Santa Clara, CA, USA) was used to collect MS data in negative ion mode. The parameters were as follows: mass range: 50–1000 *m*/*z*; dry gas temperature: 300 °C; dry gas flow rate: 5 L/min; nebulizer: 35 psi; capillary voltage: 3500 V; fragmentor: 135 V; skimmer: 65 V; full MS scan at a resolution of 20,000 was used. Two scans were obtained per s to provide data points for relative quantification. An MS2 scan was carried out using the data-dependent mode with a collision energy of 20 V.

### 3.4. Metabolomics Profiles and MS Data Analysis

High-resolution full-scan MS and MS/MS data were analyzed using MassHunter qualitative analysis software (Agilent) (Agilent Technologies Inc., Palo Alto, CA, USA) and XCMS online (https://xcmsonline.scripps.edu/, Accessed 14.12.18). The original data files (.d) were converted to mzXML format using ProteoWizard (ProteoWizard, Palo Alto, CA, USA). Then, the mzXML files’ data of each group were loaded and analyzed by XCMS using a standard XCMS workflow. Parameter settings for XCMS processing were as follows: minimum peak width = 10 s, maximum peak width = 60 s, mzwid = 0.015, minfrac = 0.5, bw = 5 and signal/noise threshold = 6. Then, the peak detection, retention time correction, chromatogram alignment and statistical evaluation were performed. The results contained a peak list with *m*/*z* values, peak intensity fold changes, statistical significances (*p* value), retention times and extracted peak intensities. These results were used for the subsequent multivariate statistical analysis.

A principal component analysis (PCA) and orthogonal partial least square discriminant analysis (OPLS-DA) were carried out using SIMCA-P 13 software (Umetrics, Umea, Sweden) for unsupervised and supervised multivariate analyses, respectively. Variables of the data sets were pareto-scaled and mean-centered before the multivariate analysis. Parameters of the PCA and OPLS-DA, including the goodness-of-fit parameter R^2^X, the variance of the response variable R^2^Y and the predictive ability parameter Q^2^, were also calculated for model evaluation. Furthermore, a hierarchical cluster heatmap was constructed using Biomarker Cloud (http://www.biocloud.net/. Accessed 14.12.18).

Metabolites were identified and characterized based on high-resolution MS-associated methods, which included comparing the results with databases and the literature, and comparing the retention times and fragments with those of standards. Metabolites were identified using the accurate high-resolution *m*/*z* values and MS/MS fragment patterns. First, the accurate *m*/*z* values were searched against compound databases KNApSAcK (http://kanaya.naist.jp/KNApSAcK/. Accessed 14.12.18), BATMAN-TCM (http://bionet.ncpsb.org/batman-tcm/. Accessed 14.12.18), METLIN (https://metlin.scripps.edu/. Accessed 14.20.18) and HMDB (http://www.hmdb.ca/. Accessed 14.12.18), with a mass accuracy of 10 ppm, a list of chemical formulae was generated. Then, the MS/MS spectra were analyzed by comparing both fragment patterns and isotope ratios to identify the metabolites [44].

## 4. Conclusions

In this study, the tissue-specific metabolite variations in *Polygonum cuspidatum* were investigated using an LC-MS-based metabolomics approach for the first time. A total of 53 bioactive compounds were identified, 8 for the first time in this species. In addition, the relative abundance levels of these compounds in different tissues were also measured. The phytochemical composition variations of different *P. cuspidatum* tissues clearly demonstrated that the roots and rhizomes generally contained the greatest amounts of stilbenes and anthraquinones compared with other tissues, while the flowers and leaves contained more flavonoids. In addition, 13 compounds were successfully identified, screened and quantified as potential chemical markers for the discrimination of different *P. cuspidatum* tissues. This study provides chemical bases for the distinct usage of different tissues of *P. cuspidatum* and could offer some guidance for the further exploitation of this species.

## Figures and Tables

**Table 1:** Characteristic fragment ions, retention times and *m*/*z* of metabolites tentatively identified based on reference standard, published literature, METLIN or HMDB database.

Identification	Precursor Ion	Molecular Formula	Predicted *m*/*z*	Measured *m*/*z*	PPM	RT	ms2 Fragment Ions at Negative Mode	References
Tartaric acid	[M − H]^−^	C_4_H_5_O_6_	149.0086	149.0082	2.68	0.72	130.9882(2.3), 105.0215(5.60),103.0071(1.41), 87.0133(36.37), 72.9965(100)	HMDB, METLIN
Malic acid	[M − H]^−^	C_4_H_5_O_5_	133.0137	133.0133	3.01	0.72	115.0076(19.99), 89.0280(7.69), 72.9970(37.65), 71.0176(100),59.0163(7.95)	HMDB, METLIN
Citric acid	[M − H]^−^	C_6_H_7_O_7_	191.0192	191.0201	4.71	0.80	173.0125(3.75), 111.0125(100), 87.0126(65.85), 85.0336(76.01), 67.0217(21.87), 57.0419(17.03)103.0106	HMDB, METLIN
Gallic acid	[M − H]^−^	C_7_H_5_O_5_	169.0215	169.0218	1.77	0.97	125.0284(100), 107.0178 (8.69), 111.0112(3.30), 97.0326(14.68), 79.0236(23.64), 69.0375(19.23)	Standard, METLIN
6-Galloylglucose/2-Galloylglucose	[M − H]^−^	C_13_H_15_O_10_	331.0663	331.0664	0.30	1.14	241.0128(3.41), 211.0290(13.62), 169.0172(100), 151.0054(48.81), 137.0258(4.43),125.0263(43.17), 111.0117(17.48)	HMDB, [2]
Salicylic acid beta-d-glucoside	[M − H]^−^	C_13_H_15_O_8_	299.0772	299.0775	1.00	1.34	225.0779(7.62), 179.0422(46.39), 137.0294(100), 119.0522(8.48), 93.0370(10.73), 73.0299(23.70),71.0166(26.89)	HMDB, METLIN
Epiafzelechin 3-gallate	[M − H]^−^	C_22_H_17_O_9_	425.0873	425.0861	2.82	2.23	407.0737(100), 255.0812(4.98), 151.0423(36.55), 137.0292(64.75), 125.0224(11.75)	HMDB
Epigallocatechin/Gallocatechin	[M − H]^−^	C_15_H_13_O_7_	305.0661	305.0672	3.61	2.31	219.0648(56.12), 191.0689(34.03), 167.0307(52.37), 139.0480(29.64), 137.0315(30.43), 125.0235(100), 111.0429(30.40)	HMDB, METLIN
Glucocaffeic acid	[M − H]^−^	C_15_H_17_O_9_	341.0873	341.0886	3.81	2.59	221.0438, 179.0379(100), 161.0244(6.52), 151.0395(11.84), 135.0485(33.41)	HMDB
Catechin	[M − H]^−^	C_15_H_13_O_6_	289.0712	289.0712	0.00	2.64	271.0601(5.61), 245.0827(23.84), 203.0720(53.62), 151.0424(28.46), 109.0320(100)	HMDB, Standard, [15,21]
Chlorogenic acid	[M − H]^−^	C_16_H_17_O_9_	353.0852	353.0845	1.98	2.83	191.0585(100), 179.0378(5.08), 161.0270(2.69), 87.0068(2.1)	HMDB, METLIN, [19]
Epigallate catechin gallate	[M − H]^−^	C_22_H_17_O_11_	457.0771	457.0759	2.63	2.93	303.0573(100), 287.0546(15.4), 169.0170(8.76), 165.0557(12.6) 161.0138(3.66), 151.0047(75.84), 125.0271(32.68)	HMDB, METLIN,
3-p-Coumaroylquinic acid	[M − H]^−^	C_16_H_17_O_8_	337.0923	337.0931	2.37	3.29	277.0685(10.43), 231.0627(2.99), 191.0540(29.44), 173.0443(100), 163.0440(26.17), 119.0509(13.82)	HMDB
Epicatechin	[M − H]^−^	C_15_H_13_O_6_	289.0712	289.0707	1.73	3.59	289.0729(53.01), 245.0939(33.89), 221.0838(18.71), 203.0728(100), 151.0438(45.34), 123.0561(73.41), 109.0356(66.28)	HMDB, [2,21]
Apigenin-6-glucoside 8-carabinoside	[M − H]^−^	C_26_H_27_O_14_	563.1395	563.1385	1.77	4.12	563.1375(100), 545.1283(3.52), 503.1142(7.92), 473.1088(10.53), 443.0946(12.11), 383.0897(10.82), 353.0642(10.79)	HMDB, [22]
Procyanidin B1	[M − H]^−^	C_30_H_25_O_12_	577.1346	577.1371	4.33	4.36	559.1370, 535.1225(23.27), 425.0878(38.95), 289.0731(100), 125.0276(54.62)	HMDB, [2,21]
3-galloylprocyanidin B1/B2	[M − H]^−^	C_37_H_29_O_16_	729.1456	729.1429	3.70	4.42	603.1210(10.82), 577.1231(32.4), 441.0865(19.36), 407.0865(100), 289.0757(40.38), 271.0661(8.43), 125.0244(18.96)	HMDB, METLIN
Resveratrol-4′-o-d-glucopyranoside	[M + COOH]-	C_21_H_23_O_10_	435.1286	435.1290	0.92	4.67	389.1263(2.05), 227.0733(100), 185.0636(1.17), 143.0524(0.64),	[2,21]
Epicatechin gallate	[M − H]^−^	C_22_H_17_O_11_	441.0822	441.0809	2.95	4.69	303.0592(2.11),290.0796(14.52), 289.0770(47.78), 271.0629(7.1), 245.0826(8.03),169.0182(100), 151.0501(2.87), 125.0281(26.39)	HMDB, [21,22,23]
Piceid	[M − H]^−^ [M + HCOO]-	C_20_H_21_O_8_ C_21_H_23_O_10_	389.1240 435.1286	389.1236 435.1279	1.03 1.61	5.09 5.09	227.0723(100), 185.0687(5.44), 143.0526(1.28), 389.1263(2.05), 227.0733(100)	HMDB, METLIN, Standard, [21]
Rutin	[M − H]^−^	C_27_H_29_O_16_	609.1456	609.1480	3.94	5.09	609.1493(100), 343.0538(1.34), 301.0387(16.57), 300.0319(25.44), 271.0245(1.43), 255.0305(0.78), 151.0107(0.63),	HMDB, METLIN, [23]
Catechin gallate	[M − H]^−^	C_22_H_17_O_10_	441.0822	441.0809	2.95	5.23	303.0549(1.63), 289.0750(53.51), 245.0450(13.48), 205.0571(3.22), 169.0179(100),	HMDB, [22]
Quercetin 4′-glucoside	[M − H]^−^	C_21_H_19_O_12_	463.0877	463.0859	3.87	5.30	343.0383(1.45), 301.0367(100), 283.0280 (1.21), 178.9999(6.03), 151.0080(2.21), 107.0185(0.65)	HMDB, METLIN
kaempferol galactoside	[M − H]^−^	C_21_H_19_O_11_	447.0922	447.0940	4.03	5.36	285.0455(100), 284.0392(17.21), 245.088(32.35)	HMDB, [22]
Resveratrol-3-o-beta-d-(sulfonyl)-glucopyranoside	[M − H]^−^	C_20_H_21_O_11_S	469.0799	469.0776	4.70	5.48	269.0746(4.56), 227.0669(8.43), 241.0073(100)	HMDB, [2,21]
Quercetin xyloside	[M − H]^−^	C_20_H_17_O_11_	433.0771	433.0759	2.77	5.88	301.0384(100), 283.0242(1.57), 271.0315(11.66), 243.0355(1.93), 151.0068(5.46)	HMDB
Hydroxyl aloe-emodin-o-glucoside	[M − H]^−^	C_21_H_19_O_9_	447.0932	447.0949	3.80	6.03	241.0542(100), 403.1076(31.52), 197.0674(6.79), 161.0422(1.32)	[2], HMDB, METLIN, [21]
Resveratrol-3-d-(6 or 2-galloyl)glucopyranoside	[M − H]^−^	C_27_H_25_O_12_	541.1341	541.1337	0.74	6.17	541.1337(100), 417.093(9.36), 313.0581(25.1), 227.0760(10.23), 169.0157(13.53)	[2], METLIN
Procyanidin-B-1, 3-o-gallate	[M − H]^−^	C_37_H_29_O_16_	729.1450	729.1463	1.78	6.24	245.0816(19.85), 289.0719(59.57), 407.0833(100), 451.0952(26.42)	[2], HMDB
Kaempferide	[M − H]^−^	C_16_H_11_O_6_	299.0556	299.0566	3.34	6.43	283.0082(4.81), 271.0251(6.79), 213.0597(100), 151.0082(17.20)	HMDB
Apigenin 7-*O*-glucoside	[M − H]^−^	C_21_H_19_O_10_	431.0978	431.0995	3.94	6.69	431.1004(100), 311.0561(0.65), 269.0497(92.44), 240.0466(10.54)	HMDB [2], METLIN
Resveratrol	[M − H]^−^	C_14_H_11_O_3_	227.0708	227.0715	3.08	6.95	211.0384(6.59), 185.0640(100), 183.0846(10.63) 159.0824(11.21), 143.0506(80.26), 133.0355	Standard, HMDB, METLIN, [18]
Emodin-o-(sulfonyl)-glucoside	[M − H]^−^	C_21_H_19_O_13_S	511.0541	511.0547	1.17	7.21	431.1007(100), 311.0631(1.68), 270.0539(12.13), 269.0485(41.04), 257.0494(1.03), 241.0040(4.82), 225.0545(0.82)	[2], METLIN, [21]
Torachrysone 8-glucoside	[M − H]^−^	C_20_H_23_O_9_	407.1342	407.1353	2.70	7.86	245.0811(100), 269.0744(0.4)	METLIN, HMD, [21]
Luteolin	[M − H]^−^	C_15_H_9_O_6_	285.0394	285.0387	2.46	8.04	285.0381(100), 267.0342(2.78), 257.0439(1.03),243.0279(1.84), 241.0489(2.14), 229.0493, 217.0488(3.9), 175.0381(5.29), 151.0031(11.51), 133.0329(18.27), 121.0273(0.73), 107.0162(3.31)	HMDB, METLIN, [2,24]
Emodin-6-o-glucoside	[M − H]^−^	C_21_H_19_O_10_	431.0978	431.0982	0.93	8.14	431.1000(48.67), 311.0584(4.54), 293.0518(1.66), 282.0597(0.7), 269.0497(100), 225.0582(2.41),	HMDB, [21]
Torachrysone-8-o-(sulfonyl)-glucoside	[M − H]^−^	C_20_H_23_O_12_S	487.0905	487.0892	2.67	8.41	407.1355, 287.0927(1.03), 245.0841(100), 243.0051(8.32), 241.0076(90.07), 230.0601(7.32), 215.0435(0.55), 113.0251(1.32)	[2,21,22]
Hydroxyl aloe-emodin	[M − H]^−^	C_15_H_9_O_6_	285.0394	285.0387	2.46	8.93	285.0401(100), 241.0543(2.17), 211.0421(0.34), 197.0586(0.69), 195.0533(0.4), 167.05(0.33), 151.0073(10.22), 133.0319(14.97),	HMDB, [21]
6″-Malonylgenistin	[M − H]^−^	C_24_H_21_O_13_	517.0982	517.0983	019	9.05	473.1056(100), 455.0952(0.53), 431.0968(2.51), 311.0553(3.47), 269.0454(75.44), 413.0835(0.25)	[22], HMDB
Apigenin	[M − H]^−^	C_15_H_9_O_5_	269.0450	269.0449	0.37	9.10	269.0494(100), 241.0493(2.97), 227.0409(3.04), 225.0536(36.73), 151.0063(1.39),	METLIN, HMDB, [25]
Apigenin 7-*O*-(6″-*O*-acetylglucoside)	[M − H]^−^	C_23_H_21_O_11_	473.1084	473.1086	0.42	9.15	473.1107(100), 431.0898(10.92), 413.0669(10.97), 311.0626(16.45), 269.0460(37.55),	HMDB
Bergapten	[M − H]^−^	C_12_H_7_O_4_	215.0344	215.0351	3.26	9.23	171.0469(2.83), 159.0484(100), 143.0535(18.1), 131.0531(86.87)	HMDB, METLIN
Torachrysone-8-o-(acetyl)-glucoside	[M − H]^−^	C_22_H_25_O_10_	449.1442	449.1439	0.45	9.24	269.0438(2.47), 245.0821(100), 230.0583(11.11), 215.0363(2.21),	[2], HMDB
Acetylemodin-o-glucoside	[M − H]^−^	C_23_H_21_O_11_	473.1078	473.1073	1.06	9.54	335.9747(3.94), 311.0626(16.45), 283.0506(5.9), 269.0460(100), 267.07(12.37)	[2,21]
Physcion-8-glucoside	[M − H]^−^	C_22_H_21_O_10_	445.1129	445.1145	3.59	9.71	325.0434(2.31), 283.0630(100), 240.0445(14.86), 165.0464(1.63),	[18,21]
6″-*O*-Acetylglycitin	[M − H]^−^	C_24_H_23_O_11_	487.1240	487.1240	0	11.35	283.0645(100), 267.0868(1.18)	HMDB
6-hydroxylrhein	[M − H]^−^	C_15_H_7_O_7_	299.0186	299.0187	0.33	11.48	300.0192(32.33), 299.0193(100), 255.0328(16.88), 227.0381(6.13), 211.0423(12.13), 199.0473(1.99)	[2,21]
Formononetin 7-*O*-glucoside	[M − H]^−^	C_22_H_21_O_9_	429.1186	429.1175	2.56	12.18	411.0650(7.35), 307.0586(7.87), 293.0452(100), 267.0667(21.06), 237.0614(6.34)	HMDB, METLIN
Acetylemodin	[M − H]^−^	C_17_H_11_O_6_	311.0551	311.0560	3.11	12.79	311.0563(100), 269.0429(14.3), 268.0402(48.88), 240.0466(3.84), 224.0481(1.22),196.0565(1.02)	[2,21]
Apigenin 7-sulfate	[M − H]^−^	C_15_H_9_O_8_S	349.0018	349.0034	4.58	12.81	331.2035(0.19), 306.3984(0.17), 269.0437(100), 241.0124(0.71), 227.0115(0.45)	HMDB, METLIN
Emodin	[M − H]^−^	C_15_H_9_O_5_	269.0450	269.0460	3.71	15.00	241.0512(21.33), 225.0546(100), 197.0626(1.11), 181.0666(1.33), 157.0626(0.39), 65.0044(0.14)	Standard, [18], HMDB, [21]
Physcion	[M − H]^−^	C_16_H_11_O_5_	283.0606	283.0611	1.77	15.39	269.0381(24.64), 268.0362(100), 241.0456(8.91), 240.0412(21.34), 205.0512(13.4), 197.2618(1.65)	METLIN, [21]

## Supplementary Materials

The following are available online. Figure S1: Typical LC-MS extracted ion chromatograms of metabolites that extracted from different tissues.
