# Supplementary material for: A Study on Tissue-Specific Metabolite Variations in Polygonum cuspidatum by High-Resolution Mass Spectrometry-Based Metabolic Profiling
Source: Molecules. 2019 Mar 18;24(6):1058. doi: 10.3390/molecules24061058 (PMC6471859; doi:10.3390/molecules24061058)
Supplement: Supplementary file 1 [file molecules-24-01058-s001.pdf]

Figure S1. Typical LC-MS extracted ion chromatograms of metabolites that extracted from different tissues.

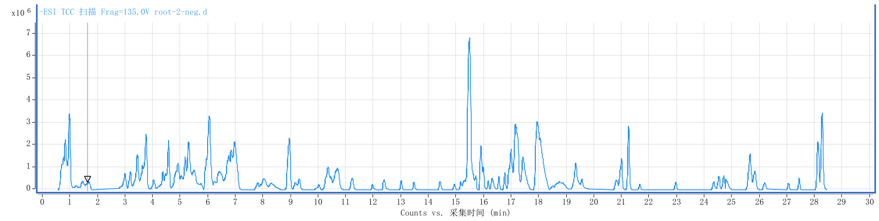

(a)

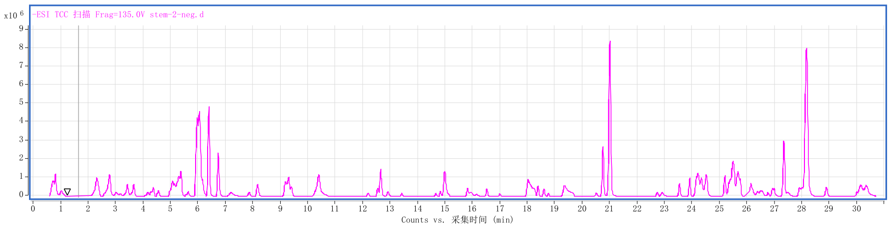

(b)

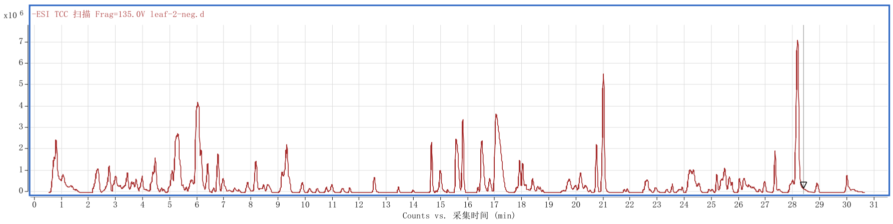

(c)

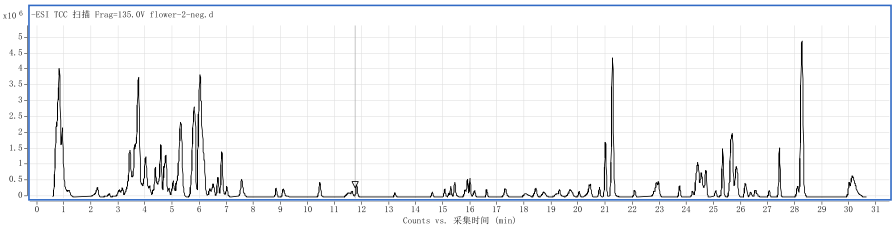

(d)

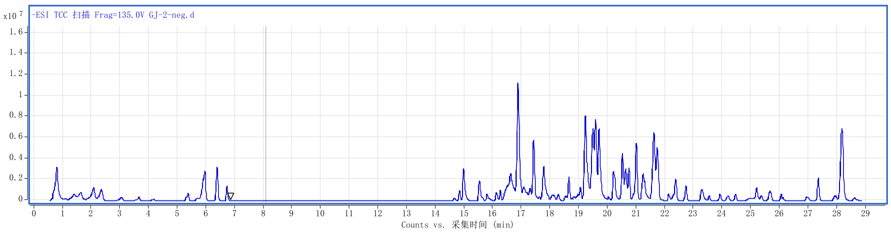

(e)

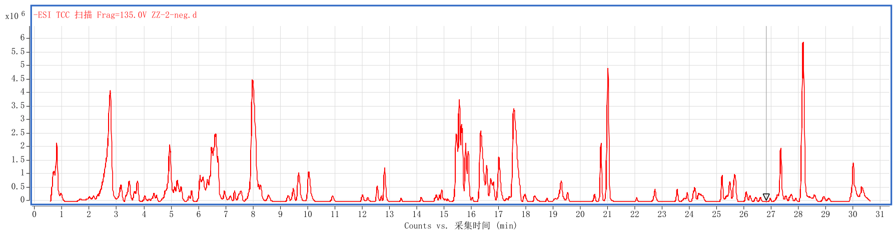

(f)

HPLC-MS (negative) total ion chromatograms (TIC) of differents parts of *P. cuspidatum*.

(a) Root, (b) Stem, (c) Leaf, (d) Flower, (e) Rhizome, (f) Seed
